# NLRP3 activation induces BBB disruption and neutrophil infiltration via CXCR2 signaling in the mouse brain

**DOI:** 10.1186/s12974-025-03468-6

**Published:** 2025-05-24

**Authors:** Jaeho Lee, Wooyoung Cho, Je-Wook Yu, Young-Min Hyun

**Affiliations:** 1 Department of Anatomy, Graduate School of Medical Science, Brain Korea 21 Project, Yonsei University College of Medicine, Seoul, Republic of Korea; 2 Department of Microbiology and Immunology, Graduate School of Medical Science, Institute for Immunology and Immunological Diseases, Yonsei University College of Medicine, Brain Korea 21 Project, Seoul, Republic of Korea

**Keywords:** Neutrophils, Experimental autoimmune encephalomyelitis (EAE), NLR family pyrin domain-containing 3 protein (NLRP3), CXCR2, Blood-brain barrier (BBB)

## Abstract

NLRP3 is an intracellular sensor molecule that affects neutrophil functionality and infiltration in brain disorders such as experimental autoimmune encephalomyelitis (EAE). However, the detailed molecular mechanisms underlying the role of NLRP3 in these processes remain unknown. We found that NLRP3 is crucial for neutrophil infiltration, whereas dispensable for neutrophil priming. Notably, NLRP3 activation in neutrophils induced blood-brain barrier (BBB) disruption and neutrophil infiltration into the brain via CXCL1/2 secretion and subsequent activation of the CXCL1/2-CXCR2 signaling axis. Moreover, CXCL1 and CXCL2 in the inflamed brain directly reduced Claudin-5 expression, which regulates BBB permeability in brain endothelial cells. Furthermore, neutrophil-specific NLRP3 activation aggravated EAE pathogenesis by promoting CXCR2-mediated infiltration of both neutrophils and CD4^+^ T cells into the central nervous system at disease onset. Thus, the CXCL1/2-CXCR2 axis plays a role in EAE progression. Therefore, this chemokine axis could be a potential therapeutic target for attenuating neuroinflammatory diseases through modulating of neutrophil and CD4^+^ T cell infiltration and BBB disruption.

**Supplementary Information:**

The online version contains supplementary material available at 10.1186/s12974-025-03468-6.

## Introduction

Neutrophils are the most abundant cell population in human blood and respond first to acute inflammation sites [1, 2]. In response to infection and injury, neutrophils migrate into the inflamed interstitial tissue where they perform antimicrobial functions such as degranulation, phagocytosis, and formation of neutrophil extracellular traps (NETs) [1, 3]. Additionally, they produce cytokines and modulate the activity of neighboring cells [4]. Neutrophil infiltration occurs in several brain-related diseases including traumatic brain injury [5], multiple sclerosis [6], ischemic stroke [7], and Alzheimer’s disease [8, 9]. Although neutrophils perform indigenous defensive immune functions as an innate immunity-associated cell population, neutrophil depletion attenuates neurological deficits and infarct volume in ischemic stroke [10], improves cognitive function in Alzheimer’s disease [8], and mitigates the severity of experimental autoimmune encephalomyelitis (EAE) [11], which highlights the potential benefits of this intervention in various brain diseases. Therefore, the factors that contribute to neutrophil infiltration in inflamed brains need to be critically investigated.

During inflammation, neutrophil infiltration generally follows a sequential multistep pathway that includes tethering, rolling, adhesion, and crawling [2]. The subsequent stage is transendothelial migration across the blood–brain barrier (BBB), which involves the paracellular [12] or transcellular pathways [13]. BBB disruption coincides with neutrophil infiltration in the inflamed brain [14, 15]. Additionally, neutrophils increase vascular permeability, as evidenced by the finding that activated neutrophils release several cytokines and chemokines that directly modulate endothelial cell permeability [16, 17]. However, the detailed mechanisms underlying the interplay between neutrophils and vascular damage remain unclear.

The NOD-like receptor family pyrin domain-containing 3 (NLRP3) is the most extensively studied intracellular sensor molecule. It recognizes a broad range of endogenous danger signals, microbial patterns, and environmental irritants, which leads to the formation and activation of the NLRP3 inflammasome [18]. NLRP3 affects the functionality and recruitment of neutrophils to inflamed tissue [19, 20]. For example, in bacteria-infected mouse brain, a lack of NLRP3 reduced neutrophil recruitment [21]. However, the precise molecular mechanism underlying NLRP3 inflammasome in NLRP3-dependent neutrophil infiltration into the brain remains unclear. Hence, in this study, we aimed to elucidate the mechanisms associated with NLRP3, BBB disruption, and neutrophil infiltration and their impact on the progression of brain disease.

## Materials and methods

### Mice

The following mouse types were used in this study: C57BL/6 mice (Orient Bio, Gyeonggi-do, Republic of Korea); *Ela*^*Cre*^ mice (EMMA #EM00075; European Mouse Mutant Archive, Monterotondo, Italy); *LysM*^*GFP*^ mice (MMRRC #012039; Mutant Mouse Resource and Research Center, CA, USA); *Nlrp3*^*D301NneoR*^ mice (JAX #017971; Jackson Laboratory, ME, USA). In *Nlrp3*^*D301NneoR*^ mice, a neomycin cassette is inserted into intron 2 of the *Nlrp3* gene at an orientation that is opposite to that of the gene, which suppresses gene expression. Additionally, these mice exhibit a point mutation in exon 3, which causes a missense mutation (D301N) that results in a gain-of-function in NLRP3. This mutation is commonly found in humans in cryopyrin-associated periodic syndrome. To induce neutrophil-specific expression of the NLRP3 (D301N) mutant [22, 23], the *Nlrp3*^*D301NneoR*^ and *Ela*^*Cre*^ mice were crossed to produce *Ela*^*Cre/+*^;*Nlrp3*^*D301NneoR/+*^ mice, which show a floxed-neoR deletion in the neutrophils. This enables the expression of the NLRP3 (D301N) mutant, whereas Cre-negative cells present the NLRP3^−/+^ phenotype.

To visualize neutrophils, *LysM*^*GFP*^ mice were crossed with *Nlrp3*^*D301NneoR*^. To induce neutrophil infiltration into the brain, either phosphate-buffered saline (PBS) or 0.8 mg/kg of lipopolysaccharide (LPS) (O111:B4, Sigma-Aldrich, Germany) was injected intraperitoneally into adult mice at 24-h intervals for 2 days. In some experiments, the mice were intraperitoneally injected with 0.5 mg/kg of the CXCR2 antagonist SB225002 (Sigma-Aldrich, Germany) at 24-h intervals for 7 days to block CXCR2 signaling [24]. To investigate the phenotypes of immune cells within the brain at the early stages of an inflammatory environment, we measured the expression levels of NLRP3, CD11b, and CD62L at 1 and 3 h post-LPS injection. The mice were maintained in a specific pathogen-free animal facility.

### Two-photon intravital microscopy

The dynamics of neutrophil migration in blood vessels or parenchyma were analyzed using two-photon intravital microscopy (TP-IVM) as described previously [25, 26]. To visualize the blood vessel structure, 10 or 70-kDa Texas red-dextran (Thermo Fisher, MA, USA) was delivered via the retro-orbital sinus. The post-capillary venules of the cortex of brain were imaged for over 20 min with 1-µm optical sections that were scanned sequentially using 1x optical zoom and a 25x objective lens with 40-µm depth. The images were analyzed using Volocity software (PerkinElmer). Each imaged brain was excited using light between 800 and 880 nm depending on the purpose. Vascular permeability was quantified as the intensity of 10-kDa of Texas Red-tagged dextran observed outside the vessel surface [26–28] (Fig. S1).

### EAE induction

The MOG_35 − 55_/CFA Emulsion PTX Kit (Hooke Labs, Lawrence, MA, USA) was used to induce EAE by following the manufacturer’s protocol. Female C57BL/6 or *Ela*^*Cre/+*^;*Nlrp3*^*D301NneoR/+*^ mice (10–12 weeks old) were immunized subcutaneously at two sites on their backs with MOG_35 − 55_ emulsified in complete Freund’s adjuvant (CFA). Subsequently, pertussis toxin (PTX) was injected intraperitoneally twice at 2 and 24-h post-immunization. Then, the EAE-treated mice were randomly allocated to each treatment group. The EAE clinical scores were assigned daily in a blinded fashion by two independent observers as follows; 0, no obvious signs of disease; 0.5, partially limp tail; 1, complete limp tail; 1.5, limp tail and waddling gait; 2, limp tail and complete paralysis of one hind limb; 2.5, limp tail, complete paralysis of one hind limb, and partial paralysis of the other hind limb; 3, limp tail and complete paralysis of both hind limbs; 3.5, limp tail, complete paralysis of both hind limbs, and ascending paralysis; 4, paralysis of trunk; 4.5, moribund; and 5, dead [29, 30].

### Single-cell dissociation from central nervous system tissue

To dissociate the cell populations in the brain and spinal cord, stock isotonic Percoll (SIP) was prepared by adding nine parts of Percoll (Percoll plus, GE Healthcare, Uppsala, Sweden) to one part of 10x Hanks’ Balanced Salt Solution (HBSS). The central nervous system (CNS) tissue of adult mice, which were transcardially perfused with 30 mL of 1x cold PBS, were homogenized. The isolated spinal cords were subjected to enzymatic treatment with 1 mg/mL collagenase D (Sigma-Aldrich) and 1 mg/mL DNase 1 (Sigma-Aldrich) at 37 °C, followed by incubation for 40 min on a shaker at 80 rpm [30]. Then, 500 mM of ethylenediaminetetraacetic acid (EDTA) was added. The CNS tissue was sieved through a 70-µm nylon mesh (SPL, Gyeonggi-do, Republic of Korea) using the plunger of a 3-mL syringe. The pellet was then resuspended in 10 mL of 30% SIP and carefully layered onto 70% SIP. The samples were then centrifuged at 500 × *g* for 30 min at 20 °C. 2–3 mL of the interface between 70% and 30% was collected [20, 31, 32].

### Flow cytometry

All cell suspensions were incubated with anti-mouse CD16/CD32 (93, Biolegend, CA, USA) to block nonspecific binding of immunoglobulin to the Fc receptors [33]. The cell surface molecules were stained in PBS containing 2% fetal bovine serum (FBS) and 2 mM EDTA for 20 min at 4 ℃. To stain intracellular molecules, the cells were permeabilized using reagents from a Foxp3/Transcription Factor Fixation/Permeabilization Kit (ThermoFisher, CA, USA) according to the manufacturer’s instructions. Intracellular cytokines were stained using the permeabilization buffer (ThermoFisher). The intravascular staining protocol was used to distinguish between vascular and tissue leukocytes, as previously described [34]. Briefly, mice were intravenously administered 3 µg of anti-CD45 antibody in 300 µL of PBS. After 3 min, the mice were perfused with cold PBS. Then, the brain cells were obtained using the single-cell dissociation protocol described earlier. The samples were visualized on an LSR II (BD Biosciences, NJ, USA) instrument and analyzed using FlowJo software (FlowJo LLC, OR, USA). All used antibodies are listed in Table S1 in Supplementary Information.

### Cranial window surgery for intravital imaging

Cranial window surgery was performed as described previously [25, 26]. Briefly, the mice were deeply anesthetized using a mixture of Zoletil (30 mg/kg) and Rompun (10 mg/kg). The mice were fixed in a stereotaxic frame (Live Cell Instrument, Republic of Korea) during all surgical procedures. Hair was removed from the frontal and parietal skull regions. A microdrill was used to create a 2-mm diameter cranial window in the right hemisphere. It was centered 2 mm lateral and 2 mm posterior to the bregma. The exposed cerebral cortex was washed with PBS and covered with a 3-mm round cover glass (Harvard Apparatus, Quebec, Canada) using tissue glue (3 M, MN, USA). A customized metal ring was fixed with dental cement (B.J.M. Laboratory, Israel) in the cranial window region to fill the imaging area with distilled water. The mouse body temperature was maintained at 37 ℃ using heating pads (Live Cell Instrument, Republic of Korea). The surgery was performed under aseptic conditions.

### Imaging data analysis

Volocity (PerkinElmer, MA, USA), Imaris (Bitplane, Switzerland), and Fiji/Image J (NIH, MD, USA) were used for 3D and 4D imaging data analyses.

## Evaluation of BBB permeability using Evans blue

The mice were intraperitoneally injected with 800 µL of 1% (w/v) Evans Blue (Sigma-Aldrich, Germany) and transcardially perfused with PBS 1 h later, as described previously [26, 35]. To quantify the Evans blue, the brain was homogenized in 1 mL of PBS and mixed with 1 mL of trichloroacetic acid (TCA). After centrifugation for 30 min at 4000 × *g*, the absorbance of the mixture was measured at 620 nm using a spectrophotometer.

### Immunofluorescence

The brains of mice were transcardially perfused with cold PBS and fixed in a 10% formalin solution (Sigma-Aldrich, Germany). The fixed brain was washed twice with PBS and placed in a 30% sucrose solution until the tissue sank to the bottom. Then, the tissue was embedded in OCT compound (Leica Biosystems, Wetzlar, Germany) and rapidly frozen in a dry ice bath. Immunofluorescence staining was performed by obtaining coronal slices (20 μm) that were blocked using an immunostaining buffer (PBS, 5% BSA, 0.5% Triton X-100) [36] to prevent non-specific antibody binding. Then, the slices were incubated with the primary antibodies overnight at 4 ℃. The slides were washed three times with PBS and stained for 1 h at room temperature using the secondary antibodies. Images were acquired using a confocal microscope (LSM710, Carl Zeiss). All used antibodies are listed in Table 1 in Supplementary Information.

### Neutrophil isolation

Mice were euthanized in a CO_2_ chamber, and bone marrow (BM) cells were obtained from the femur and tibia. The red blood cells were removed from the BM after incubation in 2 mL of ammonium–chloride–potassium lysis buffer (Gibco, NY, USA) at 25 °C for 3 min. Subsequently, the incubated cells were washed in PBS, and the cell pellets were suspended in a buffer containing 2% FBS and 2 mM EDTA in PBS. Mouse neutrophils were isolated by negative selection using a neutrophil isolation kit (Miltenyi Biotec, Bergisch Gladbach, Germany) according to the manufacturer’s instructions.

### RNA extraction and quantitative real-time PCR (qPCR)

Total RNA was prepared using the TRIzol Reagent (Invitrogen, Carlsbad, CA, USA) according to the manufacturer’s instructions. The extracted RNA was reverse transcribed into cDNA using the AccuPower CycleScript RT Premix (Bioneer, Daejeon, Republic of Korea). The mRNA expression level of each gene was measured using the SYBR Green and QuantStudio 3 systems (Applied Biosystems, Foster City, CA, USA) according to the standard protocol. All data were normalized to *Tbp* expression. All used primers are listed in Table S2 in Supplementary Information.

### In vitro culture of mouse brain endothelial cell line

The bEnd.3 cells (ATCC CRL-2299) were cultured in Dulbecco’s Modified Eagle Medium (DMEM) supplemented with 10% FBS and 1% antibiotic–antimycotic solution according to the standard protocol and maintained at 37 ℃ in a 5% CO_2_-conditioned incubator. For experimental use, cells were detached using trypsin and plated at a density of 1 × 10⁵ cells/mL in 6-well plates. After reaching confluence, the cells were stimulated with 100 ng/mL of recombinant CXCL1 and CXCL2 for 24 h [37, 38]. In some experiments, bEnd.3 cells were incubated with mouse neutrophils (1 × 10⁷ cells/well) [39] for 24 h in the presence or absence of anti-mouse CXCL1 (0.2 µg/mL, 48415, R&D Systems, MN, USA) or anti-mouse CXCL2 (2 µg/mL, 40605, R&D Systems, MN, USA) antibodies to block CXCL1 or CXCL2 [40].

### Annexin V/DAPI staining

Cell viability was assessed by incubating the cells with FITC Annexin V (Biolegend) and DAPI (1 µg/mL) solution for 15 min at 25 ℃ in the dark. The stained cells were diluted in Annexin V Binding Buffer (Biolegend), and the suspended cells were analyzed using flow cytometry.

### Enzyme-linked immunosorbent assay (ELISA)

Mouse neutrophils isolated from control and active mutant mice were lysed using PRO-PREP (Intron Biotechnology, Republic of Korea) according to the manufacturer’s instructions. Neutrophils (1 × 10⁷ cells) were lysed in 200 µL of PRO-PREP. The concentrations of chemokines were measured using CXCL1 and CXCL2 ELISA kits (R&D Systems, MN, USA) following the manufacturer’s guidelines.

### Statistical analysis

Statistical analyses were performed using Prism v9.0 (GraphPad, CA, USA). Student’s t-test or one-way ANOVA was used to compare two or more samples. The grouped samples were analyzed using a two-way ANOVA. EAE disease data was analyzed using a log-rank test that was conducted using the Mantel–Cox method. The differences were considered statistically significant at *P* < 0.05.

## Results

### Neutrophils contribute to initial inflammation in the brain through NLRP3 expression

**Fig. 1:**
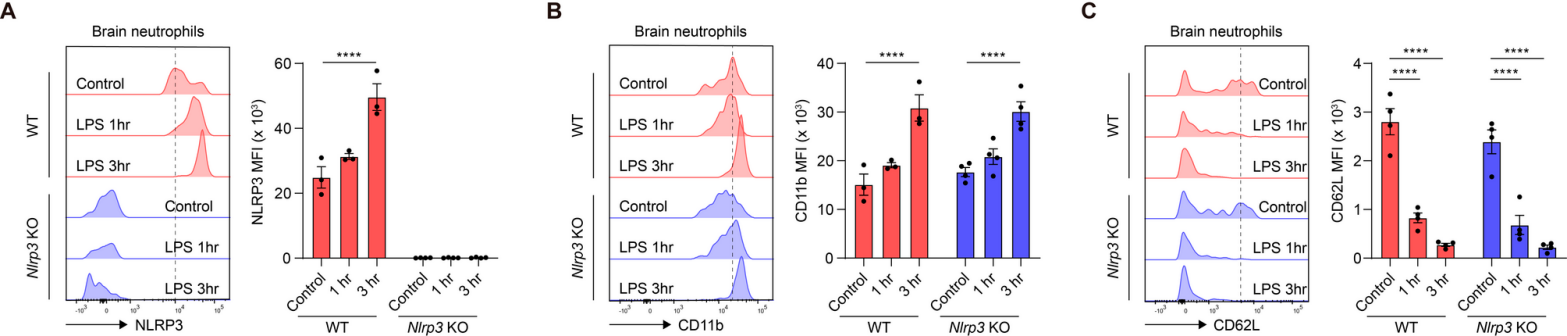
Neutrophils contribute to initial neuroinflammation through NLRP3 expression, but NLRP3 is dispensable for neutrophil priming. (**A-C**) Representative histogram and mean fluorescence intensity (MFI) of the (**A**) NLRP3, (**B**) CD11b, and (**C**) CD62L expression in neutrophils in the brain. The dotted line defines the histogram peak in the control group of WT mice. Data represent the results of at least three independent experiments. Mean values are shown with error bars representing the SEM. ****P < 0.0001

NLRP3 inhibition attenuates neutrophil infiltration in the inflamed brain [21, 41–43]. NLRP3 is expressed by various immune cells such as monocytes, microglia, and neutrophils during neuroinflammation [19, 44]. To further investigate the primary cell types potentially involved in the NLRP3-dependent neuroinflammation, we assessed NLRP3 expression levels and cellular activity in different immune cell types from brain at the early stages of bacterial LPS-induced inflammatory environment, a commonly used model of inflammation [45]. The expression levels of NLRP3 and CD11b were measured in neutrophils, monocytes, and microglia using flow cytometry at 1 and 3 h post-LPS injection in WT or *Nlrp3*^−/−^ mice. First, we define these immune cell populations by gating CD11b^+^ Ly6G^+^ neutrophils, CD45^lo/int^ CD11b^+^ microglia, and Ly6C^hi^ and Ly6C^low^ monocytes as described previously [46] (Fig. S2). WT neutrophils exhibited elevated NLRP3 and CD11b expression at 3 h post-LPS injection compared with that of control (Fig. 1A and B). In contrast, no significant differences were observed in the expression levels of these molecules in WT Ly6C^hi^, Ly6C^low^, and microglia (Fig. S3A and B). The association of CD11b with the primed phenotype in these immune cell types [20, 47–49] implies that neutrophils may contribute to initial inflammation in the brain by elevating NLRP3 expression and acting as first responders to bacterial LPS. In *Nlrp3*^−/−^ mice, NLRP3 was not detected in neutrophils as was expected (Fig. 1A). Notably, neutrophils in *Nlrp3*^−/−^ mice exhibited elevated the expression of CD11b and CD62L at 3 h post-LPS injection, which implies that NLRP3 is not necessary for neutrophil priming (Fig. 1B and C). Taken together, these findings highlight the need to further investigate the effects of NLRP3 in neutrophils during neuroinflammation.

### Neutrophil-specific NLRP3 activation facilitates neutrophil infiltration and BBB disruption

**Fig. 2:**
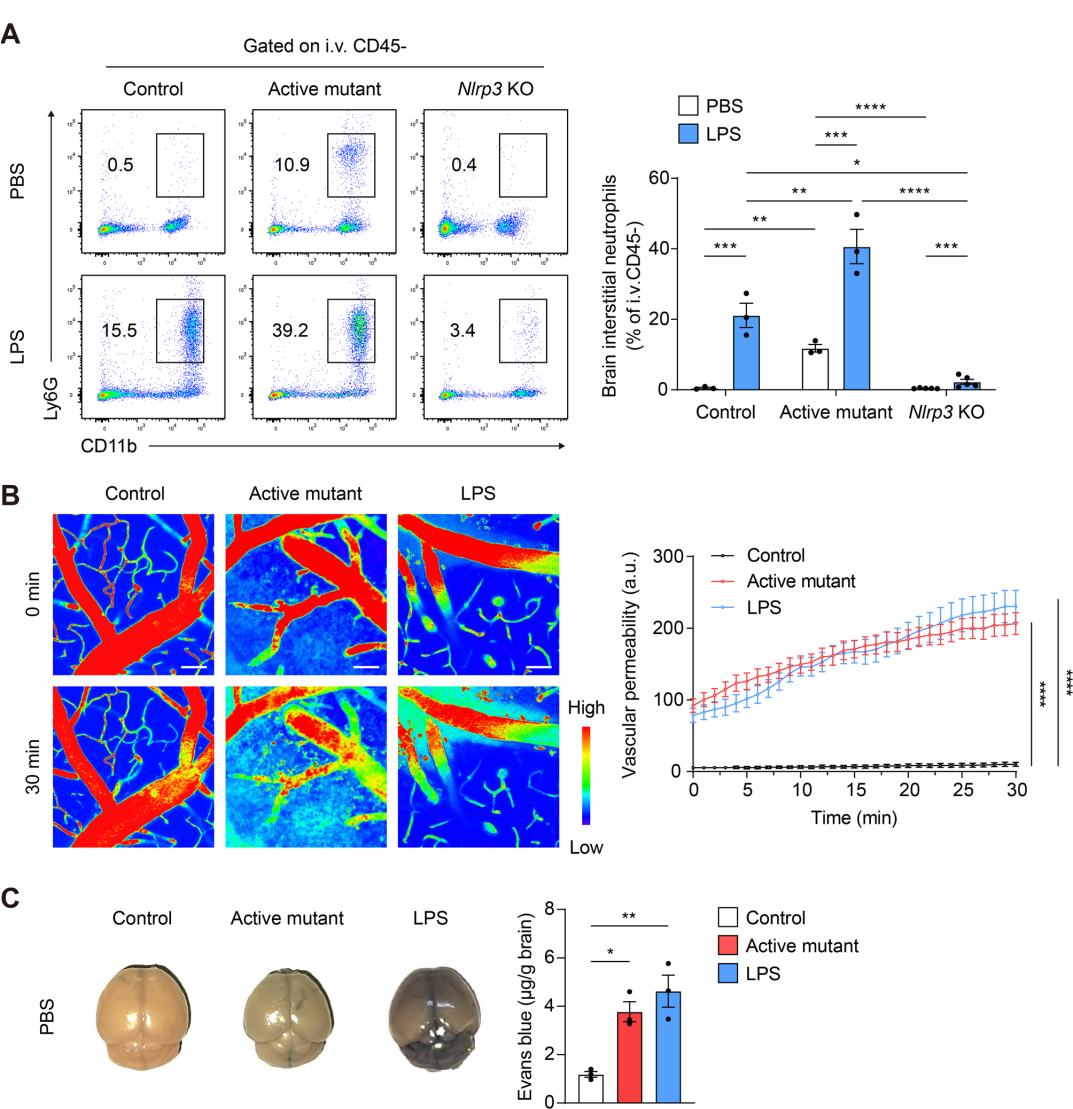
NLRP3 activation in neutrophils facilitates neutrophil infiltration and increases vascular permeability by reducing BBB integrity. (**A**) Representative scatter plots and percentage of interstitial neutrophils in the brain. *i.v.* CD45: intravenously injected anti-mouse CD45 antibody. (**B**) Two-photon intravital imaging of mice brain was performed by administering *i.v.* injection of 10 kDa Texas Red-dextran. Rainbow intensity scale was used to denote 10 kDa dextran leakage. Representative two-photon intravital images and graph of vascular leakage (extravascular dextran) at 0 min (upper panels) and 30 min (lower panels). Vascular leakage was quantified by calculating the intensity of dextran outside the venules at 1 min intervals. (**C**) Representative brain images and quantification of Evans blue in the brain. Data represent the results of at least three independent experiments. Mean values are shown with error bars representing the SEM. *P < 0.05, **P < 0.01, ***P < 0.001, ****P < 0.0001

Given that neutrophils may contribute to triggering inflammation in brain in a NLRP3 dependent manner, we further determined how NLRP3 activation in neutrophils affects their infiltration to the brain tissue and impacts BBB integrity using a mouse model of NLRP3 active mutant (D301N), which is a constitutively active form of NLRP3 [22, 23]. Active mutant mice were bred with Ela-Cre mice to induce neutrophil-specific expression of the NLRP3 active mutant. Ela-Cre caused the deletion of the floxed neomycin cassette in neutrophils of *Ela*^*cre/+*^;*Nlrp3*^*D301N/+*^ mice, leading to the expression of NLRP3 active mutants in neutrophils but not in other cells (Fig. S4A and B). In the PBS injection group, control mice exhibited minimal neutrophil infiltration, whereas active mutant mice showed a conspicuous increase in the number of infiltrated neutrophils within the brain (Fig. 2A). Following LPS injection, a higher number of infiltrated neutrophils was observed in active mutant mice compared to control mice, whereas a lower number was observed in *Nlrp3* KO mice compared to both control and active mutant mice. These findings indicate that neutrophil infiltration into the brain is dependent on NLRP3 (Fig. 2A). Here, we focused on an increase in infiltrated neutrophils induced by neutrophil-specific NLRP3 activation, in accordance with previous studies [23, 50]. Next, we investigated the effect of active mutant on BBB disruption in vivo by elucidating the expression levels of junctional proteins in the brain tissue sections pertaining to PBS-injected control and active mutant mice. The active mutant mice showed decreased Claudin-5 and ZO-1 expression (Fig. S5A and B). Additionally, intravital imaging using 10 kDa dextran to evaluate vascular permeability in the brain [27, 28] showed that active mutant mice showed pronounced dextran leakage into the interstitium, indicating a level of vascular permeability comparable to that observed in LPS-injected WT mice (Fig. 2B, Supplementary Video S1, Supplementary Video S2, and Supplementary Video S3). Furthermore, results of BBB permeability evaluation using Evans blue [26, 35] corroborated the results of the vascular permeability data obtained through TP-IVM that NLRP3 activation in neutrophils increased vascular permeability (Fig. 2C). Thus, we found that neutrophil-specific NLRP3 activation induces neutrophil infiltration and causes BBB disintegration, resulting in increased vascular permeability.

### CXCL1 and CXCL2 reduce extracellular Claudin-5 and VE-Cadherin levels in brain endothelial cells

**Fig. 3:**
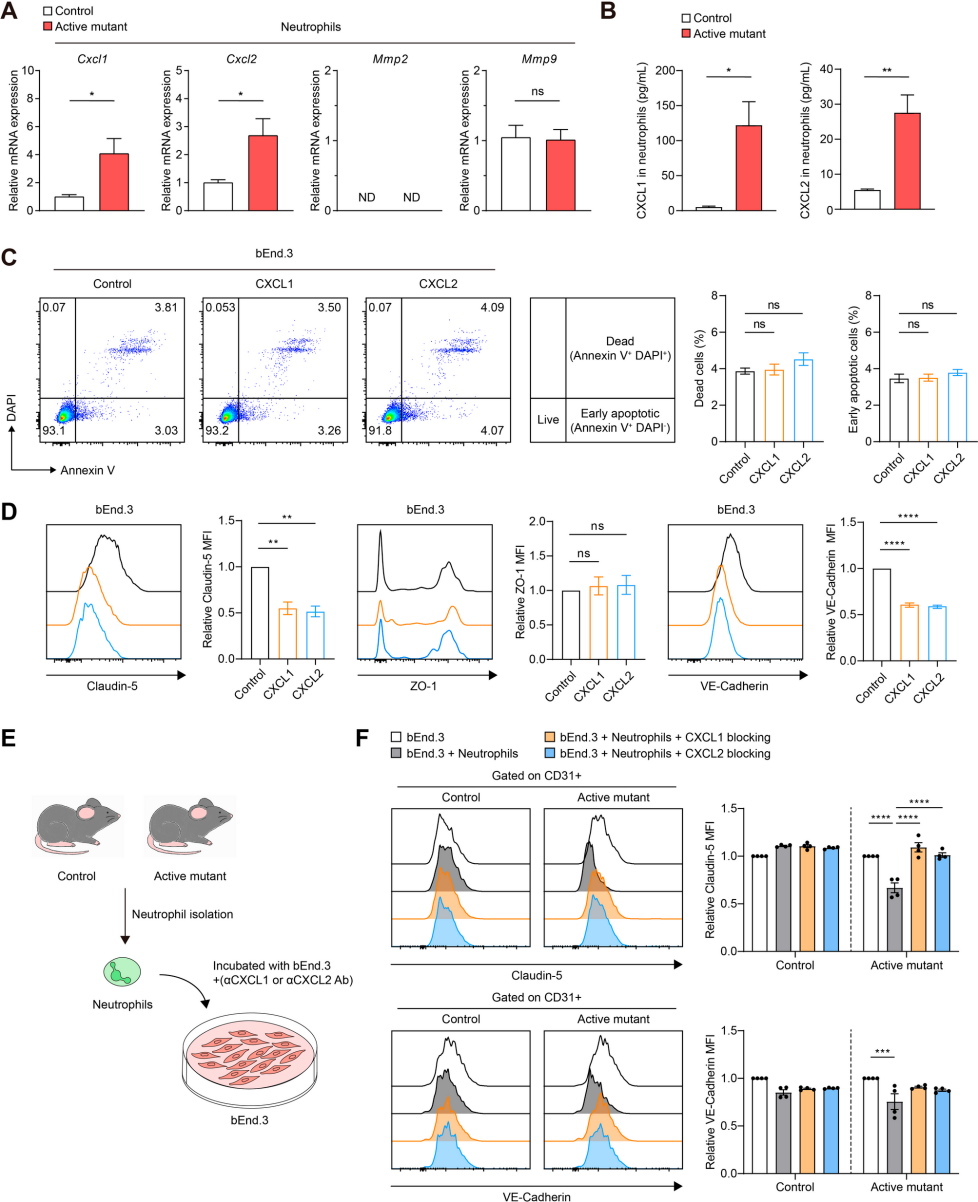
NLRP3-activated neutrophil-induced CXCL1 and CXCL2 expression reduces extracellular Claudin-5 and VE-Cadherin on brain endothelial cells. (**A**) Total RNA was isolated from neutrophils in the bone marrow of mice, and *Cxcl1*, *Cxcl2*, *Mmp2*, and *Mmp9* expression levels were analyzed using quantitative real-time PCR (qPCR). ND: not detectable. (**B**) ELISA analysis for CXCL1 and CXCL2 in the neutrophils. (**C**) Representative scatter plots and percentage of dead cells and early apoptotic cells in the brain endothelial cells. (**D**) Representative histograms and mean fluorescence intensity (MFI) of extracellular Claudin-5 (left), intracellular ZO-1 (center), and extracellular VE-Cadherin (right) in brain endothelial cells. (**E**) Experimental scheme for co-culturing neutrophils and bEnd.3 cells. Neutrophils were isolated from the bone marrow of control and active mutant mice. bEnd.3 cells were then stimulated with the isolated neutrophils in the presence or absence of anti-CXCL1 or anti-CXCL2 blocking antibodies. (**F**) Representative histograms and mean fluorescence intensity (MFI) of Claudin-5 (upper panels) and VE-Cadherin (lower panels). Data represent the results of at least three independent experiments. Mean values are shown with error bars representing the SEM. ns: non-significant. *P < 0.05, **P < 0.01

Next, we identified the potential mediator between NLRP3 activation in neutrophils and BBB disruption. Initially, the *Cxcl1*, *Cxcl2*, *Mmp2*, and *Mmp9* mRNA expression levels were measured as these were associated with BBB disruption [15, 51, 52] to determine if NLRP3 activation in neutrophils altered the production of these molecules. Although neutrophils isolated from NLRP3 active mutant mice exhibited increased *Cxcl1* and *Cxcl2* production, no significant differences were observed in *Mmp2* and *Mmp9* production (Fig. 3A). Additionally, we corroborated that CXCL1 and CXCL2 protein expression levels were increased in NLRP3 active mutant neutrophils (Fig. 3B). BBB disruption is linked to impaired endothelium and decreased expression of tight junctions [53, 54], which indicates a potential alteration in endothelial function. Hence, we evaluated if these chemokines modulated the phenotype of the brain endothelial cells (ECs). Flow cytometry was performed to assess cellular damage and expression of junctional proteins in bEnd.3 cells, which are primary mouse brain ECs [55, 56]. Neither CXCL1 nor CXCL2 induced cell death or early apoptosis in the brain ECs (Fig. 3C). Notably, these chemokines significantly reduced the expression of Claudin-5 and VE-Cadherin but not that of ZO-1 in the brain ECs (Fig. 3D). To further examine whether NLRP3 activated neutrophils reduce these junctional proteins through CXCL1 and CXCL2, bEnd.3 cells were incubated with neutrophils from control or active mutant mice in the presence or absence of anti-CXCL1 or anti-CXCL2 blocking antibodies (Fig. 3E). Notably, active mutant neutrophils directly decreased the expression of Claudin-5 and VE-Cadherin in brain ECs, and this decrease was reversed by blocking CXCL1 and CXCL2 (Fig. 3F). Taken together, these findings show that CXCL1 and CXCL2, which are produced because of NLRP3 activation in neutrophils, reduce Claudin-5 and VE-Cadherin expression in the brain ECs.

### CXCL1 and CXCL2 are produced by astrocytes, pericytes, neutrophils, and endothelial cells

**Fig. 4:**
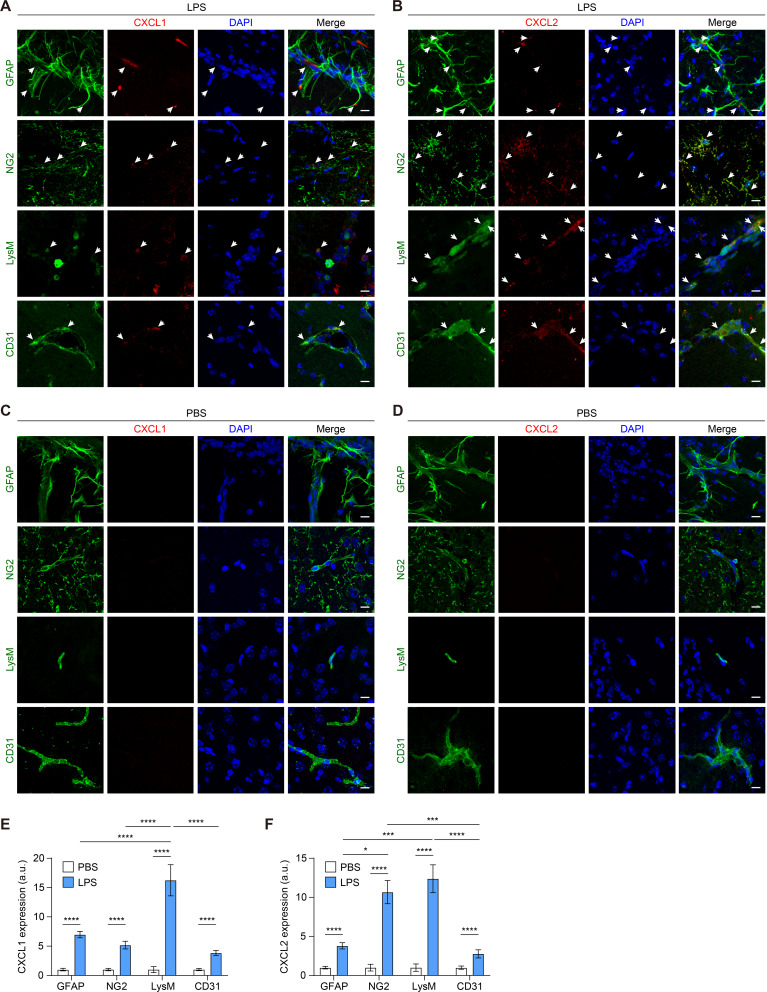
CXCL1 and CXCL2 are produced by astrocytes, pericytes, neutrophils, and endothelial cells during neuroinflammation. Immunofluorescence staining of the cerebral cortex region in coronal brain sections from (**A** and **B**) LPS-injected WT mice and (**C** and **D**) PBS-injected WT mice. Representative fluorescent images of (**A** and **C**) CXCL1 and (**B** and **D**) CXCL 2 in astrocytes, pericytes, neutrophils, and endothelial cells in the brain. Fluorescence images were acquired by staining for GFAP, NG2, LysM, or CD31 (green), CXCL1 or CXCL2 (red), and nuclei (blue). Scale bar: 10 μm. Data represent the results from the cortex region, based on the analysis of at least 15 brain slices per group across three independent experiments. Quantification of (**E**) CXCL1 and (**F**) CXCL2 levels in these cells. *P < 0.05, ***P < 0.001, ****P < 0.0001

As CXCL1 and CXCL2 reduce Claudin-5 expression in the brain ECs, we examined which cell populations were associated with CXCL1 and CXCL2 production in the brain during inflammation. To achieve maximum NLRP3 activation in the brain, WT mice were injected with LPS (0.8 mg/kg/day) at 24 h intervals for 2 days [57]. As astrocytes, pericytes, and endothelial cells are integral components of the BBB [58], we assessed the capacity of these cells and neutrophils to produce CXCL1 and CXCL2. The brain sections were stained with GFAP for astrocytes [15], NG2 for pericytes [59, 60], and CD31 for ECs [60]. LysM-GFP mice were used to identify neutrophils [15]. In the inflamed brain, CXCL1 and CXCL2 co-localized with GFAP^+^ astrocytes, NG2^+^ pericytes, LysM^+^ neutrophils, and CD31^+^ endothelial cells (Fig. 4A and B). However, in the non-inflamed brain, the expression of CXCL1 and CXCL2 was not observed in these cells (Fig. 4C and D). Moreover, the upregulation of CXCL1 was most prominent in neutrophils, while CXCL2 was markedly upregulated in both pericytes and neutrophils (Fig. 4E and F). These findings indicate that neutrophils and BBB-associated cells contribute to CXCL1 and CXCL2 production during brain inflammation.

### CXCR2 is required for neutrophil infiltration and regulation of vascular permeability in inflamed brain

**Fig. 5:**
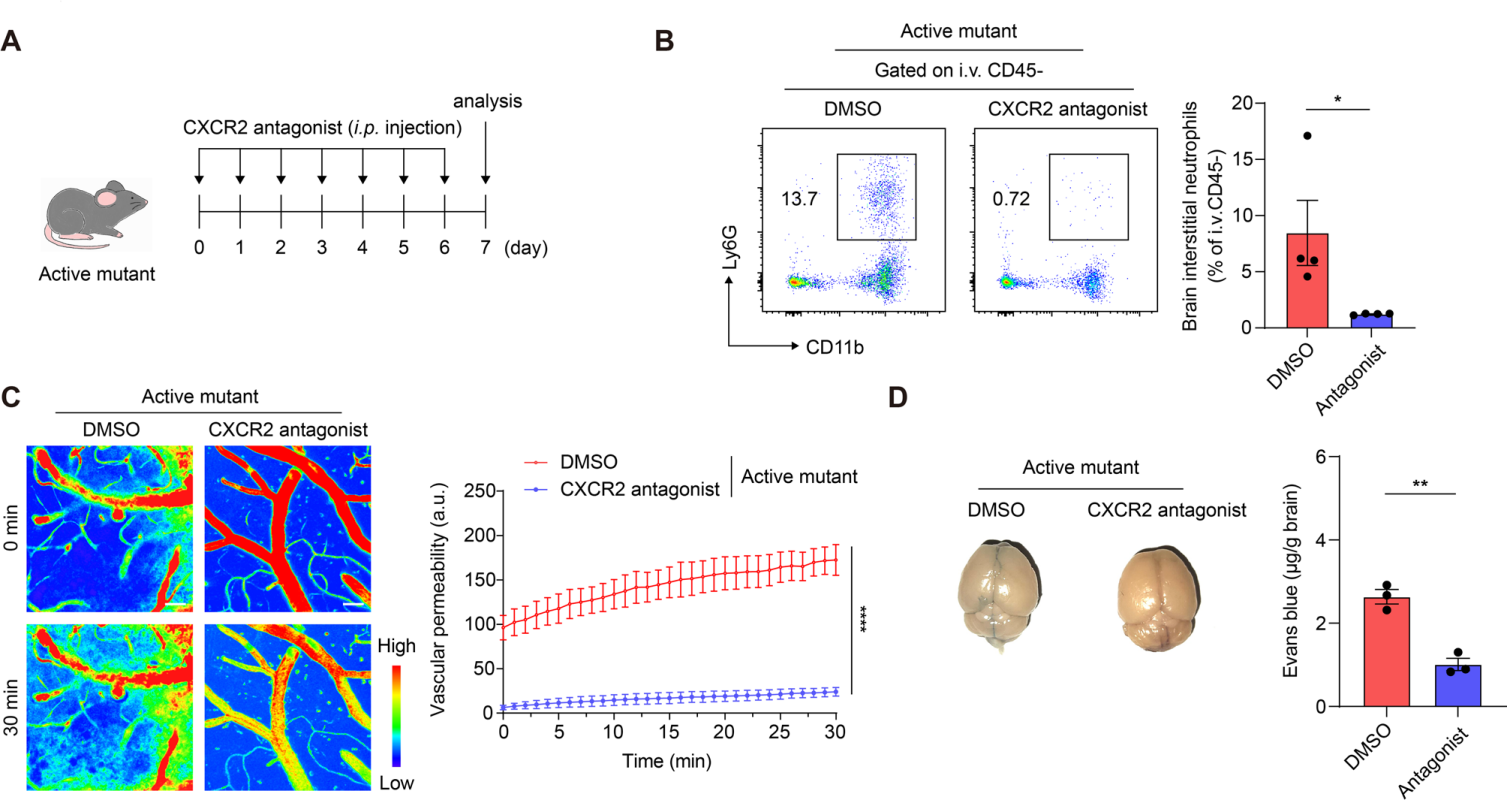
CXCR2 blockade attenuates neutrophil infiltration and increases vascular permeability. (**A**) Experimental scheme for CXCR2 injection. (**B**) Representative scatter plots and percentage of interstitial neutrophils in the brain of active mutant mice. *i.v.* CD45: intravenously injected anti-mouse CD45 antibody. (**C**) Two-photon intravital imaging of mice brains was performed with *i.v.* injection of 10 kDa Texas Red-dextran. Rainbow intensity scale was used to denote 10 kDa dextran leakage. Representative two-photon intravital images and graph of vascular leakage (extravascular dextran) at 0 min (upper panels) and 30 min (lower panels). Vascular leakage was quantified by calculating dextran intensity outside the venules at 1 min intervals. The data were obtained from independent experiments with three mice per experimental group. (**D**) Representative brain images and quantification of Evans blue in the brain of active mutant mice. Data represent the results of at least three independent experiments. Mean values are shown with error bars representing the SEM. *P < 0.05, **P < 0.01, ****P < 0.0001

We determined the effects of chemokine signaling pathways on neutrophil infiltration into the brain by blocking the cognate receptors for CXCL1 and CXCL2 in mice using a selective CXCR2 antagonist. NLRP3 active mutant mice received intraperitoneal injections of the antagonist at 24 h intervals for 7 days [24, 61] (Fig. 5A). We found that the CXCR2 blockade significantly reduced the number of interstitial neutrophils in the brain but exerted no effect on blood neutrophils in the active mutant (Fig. 5B and Fig. S6). Moreover, the CXCR2 blockade effectively restored BBB permeability in the inflamed brain (Fig. 5C, D, Supplementary Video S4, and Supplementary Video S5). We have previously reported that both neutrophil infiltration and BBB disruption are mediated by CXCR2 in the WT brain during LPS-induced inflammation [26]. Taken together, these findings suggest that CXCR2 is essential for modulating neutrophil infiltration and BBB permeability in inflamed brains.

### Neutrophil-specific NLRP3 activation aggravates EAE pathogenesis by enhancing CXCR2-mediated infiltration of both neutrophils and CD4^+^ T cells to CNS at disease onset

**Fig. 6:**
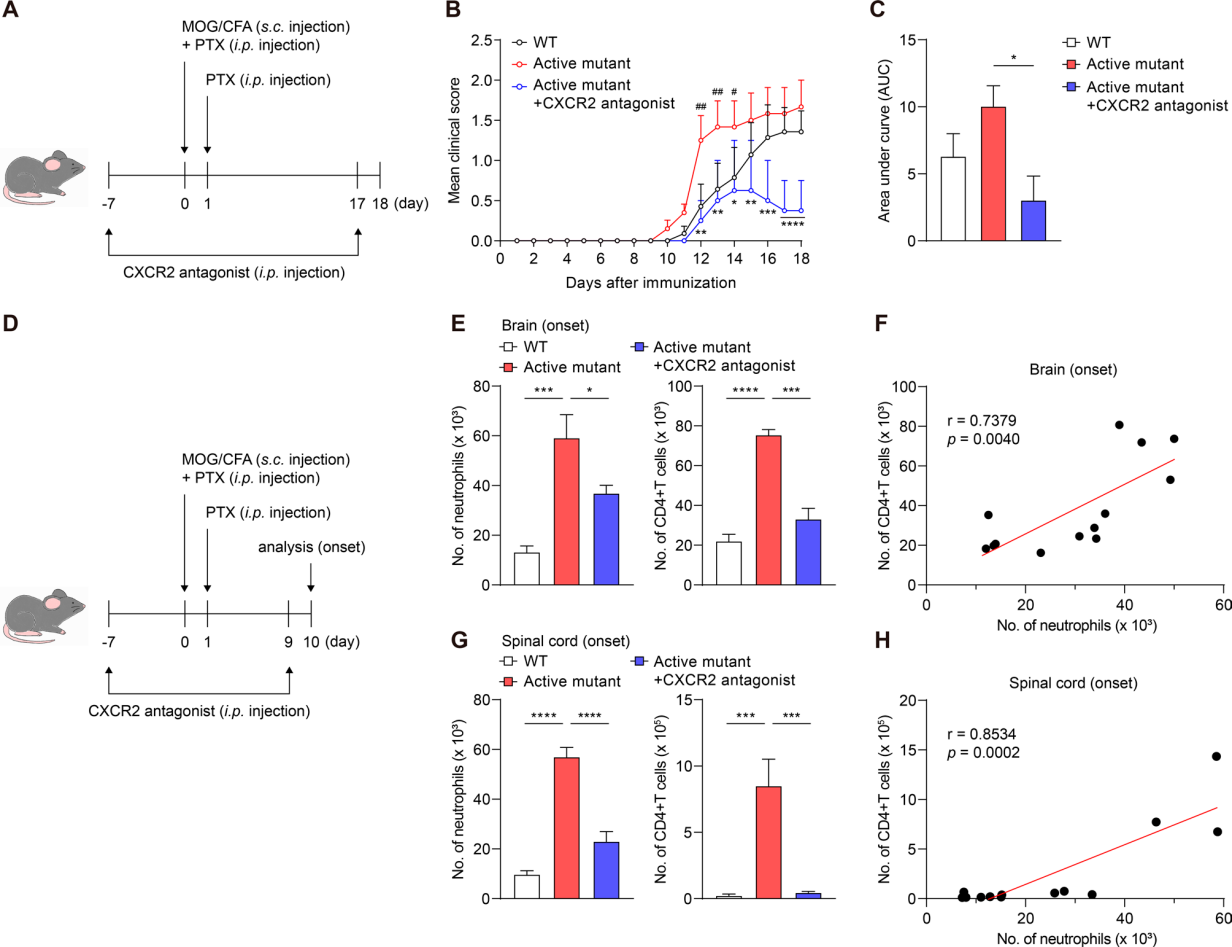
NLRP3 activation in neutrophils increased clinical score of EAE in a CXCR2-dependent manner. (**A–C**) CXCR2 antagonist or vehicle were administered daily starting from 7 days before immunization with MOG_35 − 55_ peptide antigen. EAE was induced in WT (*n* = 12), Active mutant (*n* = 10), and Active mutant + CXCR2 antagonist (*n* = 9) groups across three independent experiments. (**A**) Experimental scheme used to induce experimental autoimmune encephalomyelitis (EAE). (**B**) Mean clinical score and (**C**) quantification of the area under the curve (AUC). Number signs indicate a significant difference between the WT and Active mutant groups. Asterisks indicate a significant difference between the Active mutant and Active mutant + CXCR2 antagonist groups. (**D–H**) Control or active mutant mice were treated daily with CXCR2 antagonist or vehicle 7 days before immunization until disease onset. (**D**) Experimental scheme for CXCR2 blockade in the EAE mouse model. Number of neutrophils and CD4^+^ T cells in (**E**) brain and (**G**) spinal cord at disease onset. Correlation between number of neutrophils and CD4^+^ T cells in (**F**) brain and (**H**) spinal cord at disease onset. Mean values are shown with error bars representing the SEM. #*P* < 0.05, ##*P* < 0.01, *P < 0.05, **P < 0.01, ***P < 0.001, ****P < 0.0001

Finally, we tested our hypothesis that CXCR2-mediated inflammation in the brain, which is driven by NLRP3 activation in neutrophils, exacerbates CNS disease severity. Given that neutrophil infiltration is critical for EAE initiation [11], we used an EAE model to test this hypothesis. CXCR2 antagonist or vehicle were administered daily starting from 7 days prior to immunization with MOG_35 − 55_ peptide antigen (Fig. 6A). The active mutant increased the clinical score of EAE compared with that observed for WT mice; however, CXCR2 antagonist negated these effects (Fig. 6B and C). These results suggest that NLRP3 activation in neutrophils exacerbates EAE pathogenesis via a CXCR2-mediated inflammatory environment. Next, as neutrophil and CD4^+^ T cell infiltration prior to EAE onset contributes to disease progression [11, 62], we quantified the number of these cells in the CNS at disease onset (Fig. 6D). The active mutant enhanced neutrophil and CD4^+^ T cell infiltration into both the brain and spinal cord. However, CXCR2 antagonist reduced the infiltration of these cells (Fig. 6E and G). Additionally, we found a positive correlation between neutrophil and CD4^+^ T cell numbers in the CNS at EAE onset, indicating a potential interaction between these cells during the early stage of EAE progression (Fig. 6F and H). These results suggest that NLRP3 activation in neutrophils enhances CXCR2-mediated neuroinflammation through the infiltration of both neutrophils and CD4^+^ T cells and increased BBB permeability, which aggravates EAE pathogenesis.

## Discussion

The two important findings of this study were that (1) neutrophils primarily contribute to initial neuroinflammation through NLRP3 expression and (2) NLRP3 activation in neutrophils leads to activation of the CXCL1/2-CXCR2 axis signaling, which facilitates neutrophil infiltration and BBB disruption, revealing that this axis is crucial for EAE progression. The results of this study add a new component to the cascade of molecular mechanisms that contributes to neutrophil infiltration in the inflamed brain.

In the present study, neutrophils exhibited elevated NLRP3 and CD11b expression levels in the early stages of neuroinflammation, whereas other immune cells such as Ly6C^hi^, Ly6C^low^, and microglia showed no changes. This suggests that initial neuroinflammation primarily occurs because of NLRP3 expression in the neutrophils. Prophylactic anti-Ly6G treatment blocked neutrophil infiltration and substantially reduced pro-inflammatory cytokine levels, whereas anti-Ly6G treatment administered 4 h post-hypoxic ischemic encephalopathy (HIE) failed to prevent neutrophil infiltration and brain damage. This highlights the pathological functions of early arriving neutrophils in inflammatory condition [63]. Furthermore, these results supported our hypothesis that neutrophils play a pivotal role in the initial stage of neuroinflammation. Additionally, NLRP3-deficient neutrophils exhibited a primed phenotype at the early stages of LPS-induced neuroinflammation but eventually failed to infiltrate the inflamed brain [26, 64], indicating that NLRP3 is dispensable for neutrophil priming but is required for neutrophil infiltration.

NLRP3 activation in neutrophils triggers neutrophil infiltration into the brain as has been reported by Stackowicz et al. [50]. They have shown that NLRP3 activation in neutrophils triggers skin inflammation, leading to the recruitment of neutrophils to the skin. Additionally, Kaufmann et al. [23]. have shown that NLRP3 activation in neutrophils induces liver inflammation, which involves inflammatory cytokine production and accumulation of neutrophils in the liver. Collectively, these findings show that NLRP3 activation in neutrophils initiates neutrophil infiltration into several organs. Moreover, NLRP3 activation in neutrophils triggers BBB disintegration, leading to increased permeability. These findings concur with those of several studies that have shown that activated neutrophils increase vascular permeability [65–68]. Furthermore, neutrophil depletion alleviates BBB breakdown in the inflamed brain [69, 70]. These findings support the hypothesis that activated neutrophils contribute to BBB disintegration, which increases vascular permeability. Taken together, NLRP3 activation in neutrophils triggers neuroinflammation, which is characterized by neutrophil infiltration into the brain and BBB disruption.

Although initially characterized as leukocyte chemoattractants, the chemokine CXCL1 and CXCL2 acted as mediators between NLRP3 activation in neutrophils and BBB disintegration in this study. The function of chemokines now extends well beyond their initial characterization as leukocyte chemoattractants [52, 71, 72]. Haarmann et al. [73]. have shown that both CXCL5 and CXCL8 disturbed the paracellular barrier function of the human brain endothelial monolayer. Yu et al. [74]. found that CXCL8 increases the permeability of the human endothelium by downregulating tight junctions. These findings support our hypothesis that these chemokines contribute to BBB dysfunction by reducing BBB integrity. In the present study, NLRP3-activated neutrophils increased CXCL1 and CXCL2 production. Additionally, both chemokines reduced the BBB integrity. This finding is consistent with the results of studies that have reported that neutrophils are the primary producers of CXCL2 and that this chemokine is critical for the precise breaching of endothelial junctions [52]. Notably, the findings of the present study show that both CXCL1 and CXCL2 reduce extracellular Claudin-5 expression, whereas they exert no effect on intracellular ZO-1 expression in the brain endothelium in vitro. This difference in Claudin-5 and ZO-1 expression may be attributed to the cellular localization of these proteins in the ECs. Taken together, these findings provide evidence that both CXCL1 and CXCL2 contribute to BBB dysfunction by reducing extracellular Claudin-5 levels in the brain endothelium.

In this study, CXCR2, which is a cognate receptor for CXCL1 and CXCL2, was found to be critical for regulating neutrophil infiltration and BBB permeability. CXCR2 blockade reduced the number of infiltrating neutrophils and vascular permeability in the inflamed brain. This finding concurs with the results of previous studies, which have reported that following LPS injection, neutrophil infiltration was reduced by blocking CXCR2 [75–77], CXCL1 [75], or CXCL2 [78]. Furthermore, BBB permeability was reduced by blocking CXCR2 [15] or CXCL1 [15] following herpes simplex virus (HSV) infection. Thus, CXCL1, CXCL2, CXCL5, and CXCL8 contribute to BBB dysfunction by reducing BBB integrity [73, 74]. Given that these chemokines are ligands of CXCR2, the CXCR2 signaling pathway plays a crucial role in regulating BBB integrity.

EAE mouse model aids in studying multiple sclerosis, which is an autoimmune demyelinating disease affecting the CNS [79, 80]. Neutrophil infiltration of the CNS at EAE onset contributes to disease progression. This is supported by the finding that neutrophil depletion prior to disease onset significantly reduces EAE severity, whereas neutrophil depletion after onset fails to alleviate disease severity [11]. The results of this study indicate that the active mutant increases the clinical score in a CXCR2-dependent manner. Moreover, the active mutant enhanced neutrophil infiltration into the CNS at EAE onset via CXCR2 signaling. In summary, our findings suggest that NLRP3 activation in neutrophils exacerbates EAE progression via CXCR2-mediated neutrophil infiltration at EAE onset.

This study has some limitations. We have concluded that CXCR2 signaling promotes neutrophil infiltration and BBB disruption in neuroinflammation models only in the scenario of NLRP3 activation in neutrophils. Therefore, further studies are needed to determine whether these findings may be generalized to other models of neuroinflammation induced by other factors. However, some studies have reported the effects of CXCR2 signaling on neutrophil infiltration and BBB disruption following HSV [15] or *S. pneumoniae* infection [81]. Thus, this study provides additional evidence supporting the essential role of CXCR2 signaling in regulating neutrophil infiltration and BBB disruption. Furthermore, the specific mechanisms underlying the interactions between neutrophils and CD4^+^ T cells in the CNS at EAE onset remain unclear. One possible underlying mechanism could be that neutrophils aid in recruiting CD4^+^ T cells to the brain through NET formation, as evidenced by findings that NET elimination reduces the number of CD4^+^ T cells in the CNS in EAE [20]. Therefore, this study provides evidence that neutrophil infiltration positively correlates with CD4^+^ T cell infiltration in the CNS during EAE onset.

In conclusion, this study shows that NLRP3 activation in neutrophils induces brain inflammation through neutrophil infiltration and BBB disruption via CXCL1/2 secretion and subsequent activation of the CXCL1/2-CXCR2 signaling axis. Additionally, the active mutant exacerbates EAE pathogenesis through CXCR2-mediated neuroinflammation, which highlights the critical role of neutrophils in EAE progression. Finally, CXCR2 signaling blockade attenuates leukocyte infiltration and BBB disruption, which alleviates EAE severity. Thus, this chemokine axis could be a potential target for therapeutic approaches to attenuate neuroinflammation by modulating neutrophil and CD4^+^ T cell infiltration and BBB disruption.

## Electronic supplementary material

Below is the link to the electronic supplementary material.

Supplementary Material 1

Supplementary Material 2

Supplementary Material 3

Supplementary Material 4

Supplementary Material 5

Supplementary Material 6

Supplementary Material 7

Supplementary Material 8

Supplementary Material 9

Supplementary Material 10

Supplementary Material 11

Supplementary Material 12

## Data Availability

No datasets were generated or analysed during the current study.

## Abbreviations

NETs: Neutrophil extracellular traps
EAE: Experimental autoimmune encephalomyelitis
BBB: Blood–brain barrier
NLRP3: NOD-like receptor family pyrin domain-containing 3
PBS: Phosphate-buffered saline
LPS: Lipopolysaccharide
TP-IVM: Two-photon intravital microscopy
CFA: Complete Freund’s adjuvant
PTX: Pertussis toxin
SIP: Stock isotonic Percoll
HBSS: Hanks’ Balanced Salt Solution
CNS: Central nervous system
EDTA: Ethylenediaminetetraacetic acid
FBS: Fetal bovine serum
TCA: Trichloroacetic acid
BM: Bone marrow
qPCR: Quantitative real-time PCR
DMEM: Dulbecco’s Modified Eagle Medium
ECs: Endothelial cells
HIE: Hypoxic ischemic encephalopathy
HSV: Herpes simplex virus
