# Supplementary material for: NLRP3 activation induces BBB disruption and neutrophil infiltration via CXCR2 signaling in the mouse brain
Source: J Neuroinflammation. 2025 May 24;22:139. doi: 10.1186/s12974-025-03468-6 (PMC12102932; doi:10.1186/s12974-025-03468-6)
Supplement: Supplementary file 1 — Supplementary Material 1 [file 12974_2025_3468_MOESM1_ESM.docx]

**Supplementary Information**

**Supplementary figure legends**

**Supplementary Figure S1. Procedure for vascular permeability analysis.**

Phase 1, The mouse brain cortex was exposed using a hand drill and then covered with a 3-mm round cover glass. A customized metal ring was affixed with dental cement around the cover glass to allow the imaging chamber to be filled with distilled water. Phase 2, Intravital imaging of the dextran-injected mouse brain was performed using a two-photon microscope. Phase 3, The intensity of dextran that leaked into the interstitial region outside the vessels was measured from the acquired images. Phase 4, Background noise intensity was subtracted from the measured dextran intensity, and the data were analyzed over time.

**Supplementary Figure S2. Gating strategy to define neutrophils, monocytes, and microglia population via flow cytometry.**

Singlets were gated to avoid the unexpected false positive signal for fluorescence labeled antibody. First, the population of negative signal for Zombie Aqua dye was gated to identify live cell population. Within live cells, the CD11b^+^ Ly6G^+^ population was denoted as neutrophils, and CD45^lo/int^ CD11b^+^ microglia were isolated from the CD11b^+^ Ly6G^-^ population. Subsequently, within the CD45^hi^ CD11b^+^ population, we gated the Ly6C^hi^ and Ly6C^low^ subsets.

**Supplementary Figure S3. At initial neuroinflammation, WT Ly6C^hi^, Ly6C^low^, and microglia show no significant changes in CD11b and NLRP3 expression levels.**

(A and B) Representative histogram and mean fluorescence intensity (MFI) of the (A) NLRP3 and (B) CD11b expression of WT Ly6C^hi^, Ly6C^low^, and microglia in the brain. Dotted line defines histogram peak in the control group of WT mice. Data represent results of at least three independent experiments. Mean values are shown with error bars representing the SEM. ns: non-significant.

**Supplementary Figure S4. Summary of NLRP3 expression in control and active mutant mice.**

(A) Summary of genotype and NLRP3 expression in neutrophils and other cell types of control and active mutant mice. (B) Schematic diagram of NLRP3 expression pattern and neomycin cassette in neutrophils and other cell types of both mice.

**Supplementary Figure S5. NLRP3 activation reduced the expression of Claudin-5 and ZO-1 in the brain.**

Representative fluorescent images and graph of (A) Claudin-5 and (B) ZO-1 level in the brain. Fluorescence images were acquired by staining Claudin-5, ZO-1 (green), and nuclei (blue). Scale bar: 20 μm. a.u.: arbitrary unit. Analysis was performed using brain slices from 3 biologically independent mice. ***P*<0.01, *****P*<0.0001.

**Supplementary Figure S6. CXCR2 antagonist exerted no effect on the number of blood neutrophils in active mutant.**

Representative scatter plots and percentages of blood neutrophils of active mutant mice. Data represent the data of at least three independent experiments. Mean values are shown with error bars representing the SEM. ns: non-significant.

**Supplementary video legends**

**Supplementary Video 1. Dextran did not leak outside the brain vessels in the control (PBS).**

Montage of compiled original red scale (left) and rainbow scale (right) images of two-photon intravital imaging of control mouse brains injected with PBS. Mice were administered an intravenous injection of 10 kDa Texas Red dextran. Brain blood vessels were imaged. Scale bar: 50 μm.

**Supplementary Video 2. BBB permeability is increased in the NLRP3 active mutant mice (PBS).**

Montage of compiled original red scale (left) and rainbow scale (right) images of two-photon intravital imaging of NLRP3 active mutant mouse brains injected with PBS. The mice were intravenously injected with 10 kDa Texas Red dextran. Brain blood vessels were imaged. Scale bar: 50 μm.

**Supplementary Video 3. BBB permeability is increased in the LPS-injected WT mice.**

Montage of compiled original red scale (left) and rainbow scale (right) images of two-photon intravital imaging of WT mouse brains injected with LPS. The mice were intravenously injected with 10 kDa Texas Red dextran. Brain blood vessels were imaged. Scale bar: 50 μm.

**Supplementary Video 4.** **BBB permeability is increased in the NLRP3 active mutant mice (DMSO).**

Montage of compiled original red scale (left) and rainbow scale (right) images of two-photon intravital imaging of NLRP3 active mutant mouse brains injected with diluted DMSO. Mice were intravenously injected with 10 kDa Texas Red dextran. Brain blood vessels were imaged. Scale bar: 50 μm.

**Supplementary Video 5. CXCR2 blockade restored BBB permeability in the NLRP3 active mutant mice.**

Montage of compiled original red scale (left) and rainbow scale (right) images of two-photon intravital imaging of NLRP3 active mutant mouse brains injected with CXCR2 antagonist. The mice were intravenously injected with 10 kDa Texas Red dextran. Brain blood vessels were imaged. Scale bar: 50 μm.

**Supplementary Table 1.** Antibody lists.

| **Antibody** | **Product information** |
| --- | --- |
| Anti-mouse CD16/CD32 | 93, Biolegend |
| PerCP anti-mouse CD45 | 30-F11, Biolegend |
| APC/cy7 anti-mouse CD45 | 30-F11, Biolegend |
| APC anti-CD11b | M1/70, Biolegend |
| BV421 anti-CD11b | M1/70, Biolegend |
| FITC anti-mouse Ly-6G | 1A8, Biolegend |
| PE anti-mouse Ly-6G | 1A8, Biolegend |
| APC/Cy7 anti-mouse Ly-6G | 1A8, Biolegend |
| Alexa Fluor 700 anti-mouse Ly-6C | HK1.4, Biolegend |
| PE anti-mouse Ly-6C | HK1.4, Biolegend |
| Alexa Fluor 700 anti-Human/Mouse NLRP3 | 768319, R&D system |
| APC anti-mouse CD62L | MEL-14, Biolegend |
| PE anti-mouse VE-Cadherin | BV13, Biolegend |
| PE anti-mouse CD18 | M18/2, Biolegend |
| APC anti-mouse CD11a | M17/4, Biolegend |
| Monoclonal anti-mouse Claudin-5 | 4C3C2, Invitrogen |
| Polyclonal anti-mouse ZO-1 | Invitrogen |
| Polyclonal anti-mouse NG | Sigma-Aldrich |
| Monoclonal anti-mouse GFAP | 2E1.E9, Biolegend |
| Monoclonal anti-mouse CD31 | 390, Biolegend |
| Monoclonal anti-mouse CXCL1 | 48415, Invitrogen |
| Polyclonal anti-mouse CXCL2 | R&D system |
| Blocking anti-mouse CXCL1 | 48415, R&D system |
| Blocking anti-mouse CXCL2 | 40605, R&D system |
| Alexa 488 anti-mouse IgG | Invitrogen |
| Alexa 488 anti-rabbit IgG | Abcam |
| Alexa 647 anti-rat IgG | Biolegend |
| Alexa 647 anti-goat IgG | Invitrogen |

**Supplementary Table 2.** Primers used for qPCR.

| *Tbp* | |
| --- | --- |
| Forward primer | GAG TTG CTT GCT CTG TGC TG |
| Reverse primer | CTG GCT TGT GTG GGA AAG AT |
| *Cxcl1* | |
| Forward primer | GCT GGG ATT CAC CTC AAG AA |
| Reverse primer | TGG GGA CAC CTT TTA GCA TC |
| *Cxcl2* | |
| Forward primer | TGG AAG GAG TGT GCA TGT TC |
| Reverse primer | CAA GAC ACG AAA AGG CAT GA |
| *Mmp2* | |
| Forward primer | CCC CTG ATG TCC AGC AAG TAG A |
| Reverse primer | AGT CTG CGA TGA GCT TAG GGA AA |
| *Mmp9* | |
| Forward primer | CCC TGG AAC TCA CAC GAC ATC TTC |
| Reverse primer | GGT CCA CCT TGT TCA CCT CAT TTT |
