# Supplementary figures and images for: NLRP3 activation induces BBB disruption and neutrophil infiltration via CXCR2 signaling in the mouse brain
Source: J Neuroinflammation. 2025 May 24;22:139. doi: 10.1186/s12974-025-03468-6 (PMC12102932; doi:10.1186/s12974-025-03468-6)

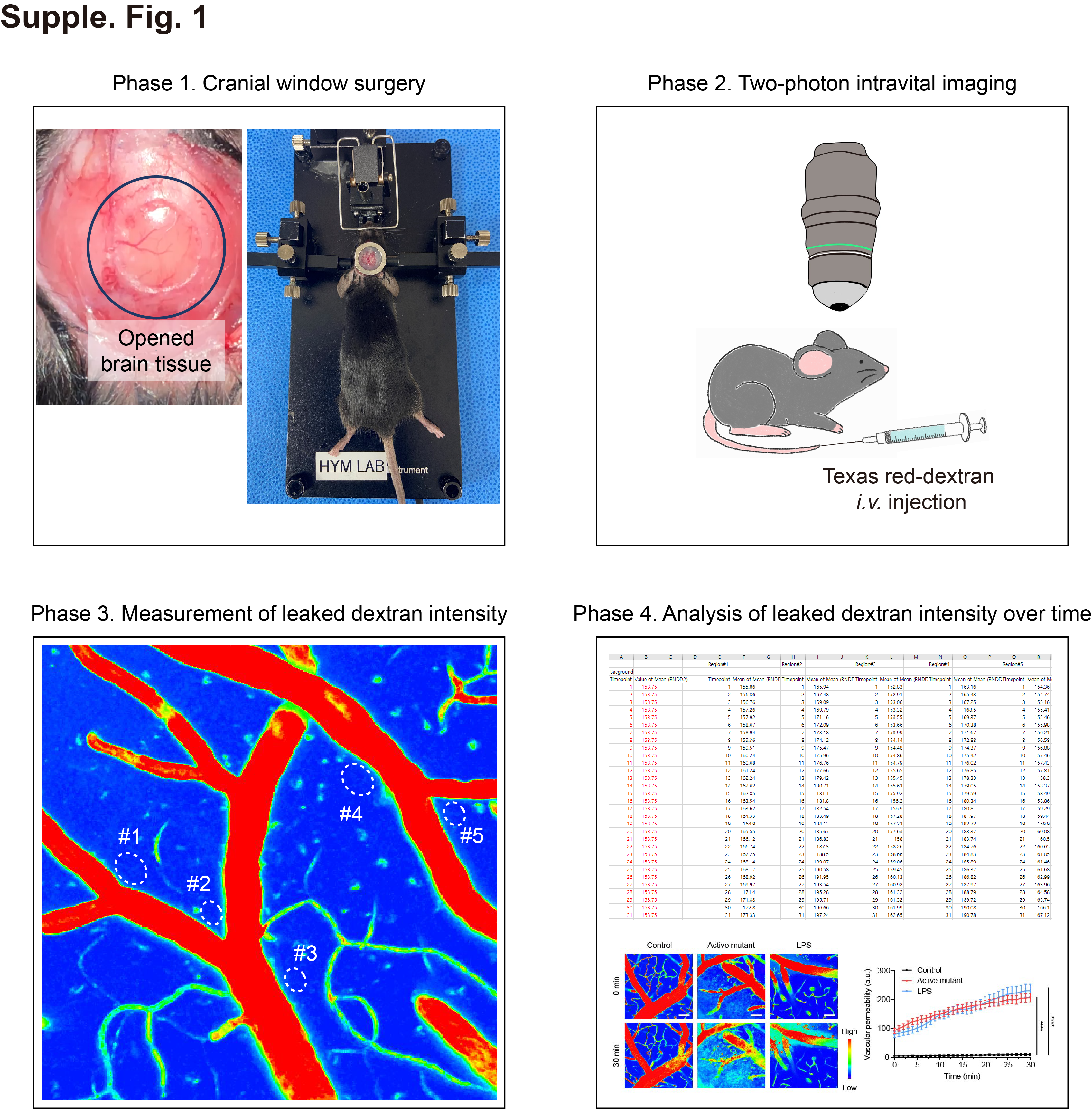

Supplement: Supplementary file 2 — Supplementary Material 2 [file 12974_2025_3468_MOESM2_ESM.png]

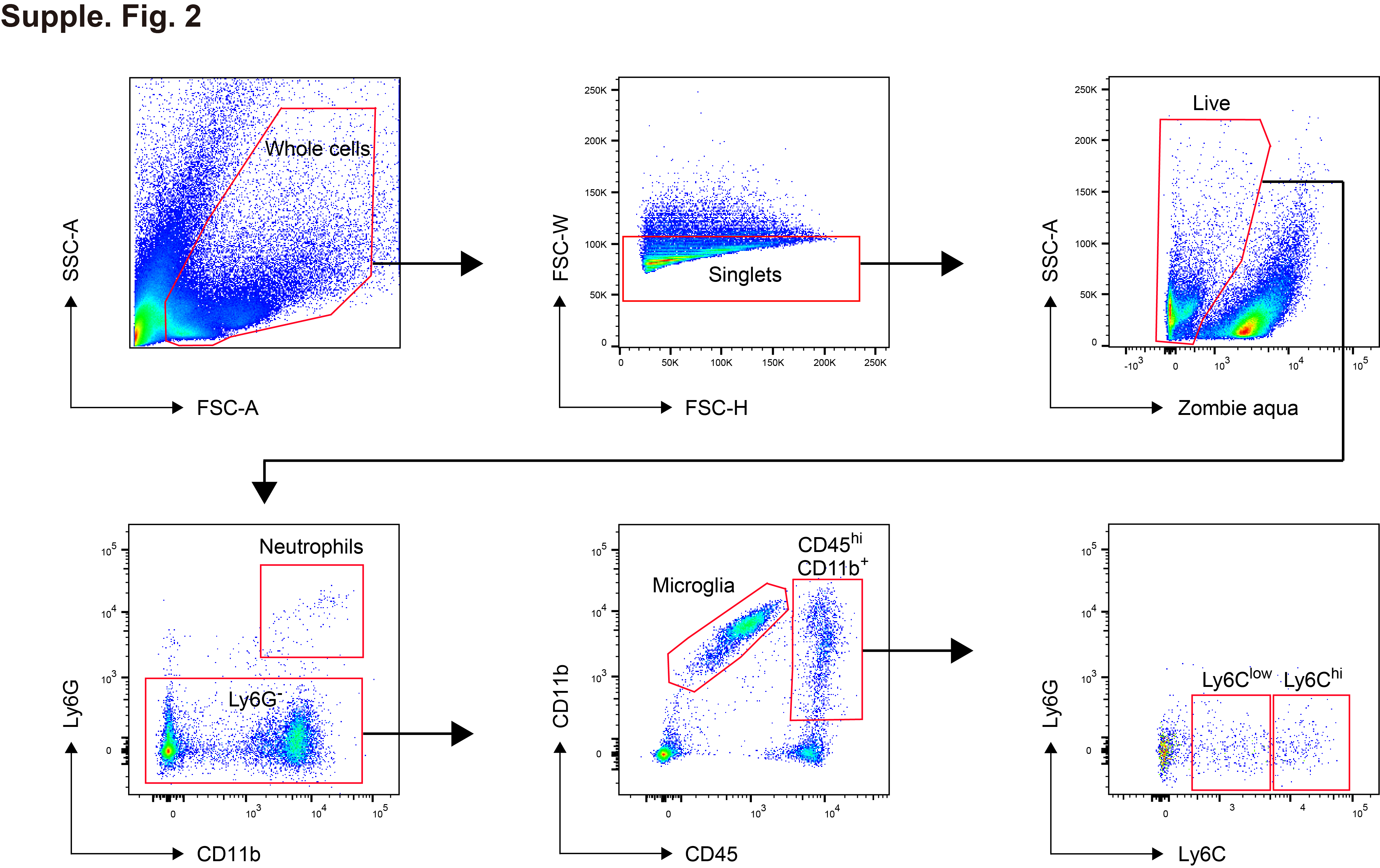

Supplement: Supplementary file 3 — Supplementary Material 3 [file 12974_2025_3468_MOESM3_ESM.png]

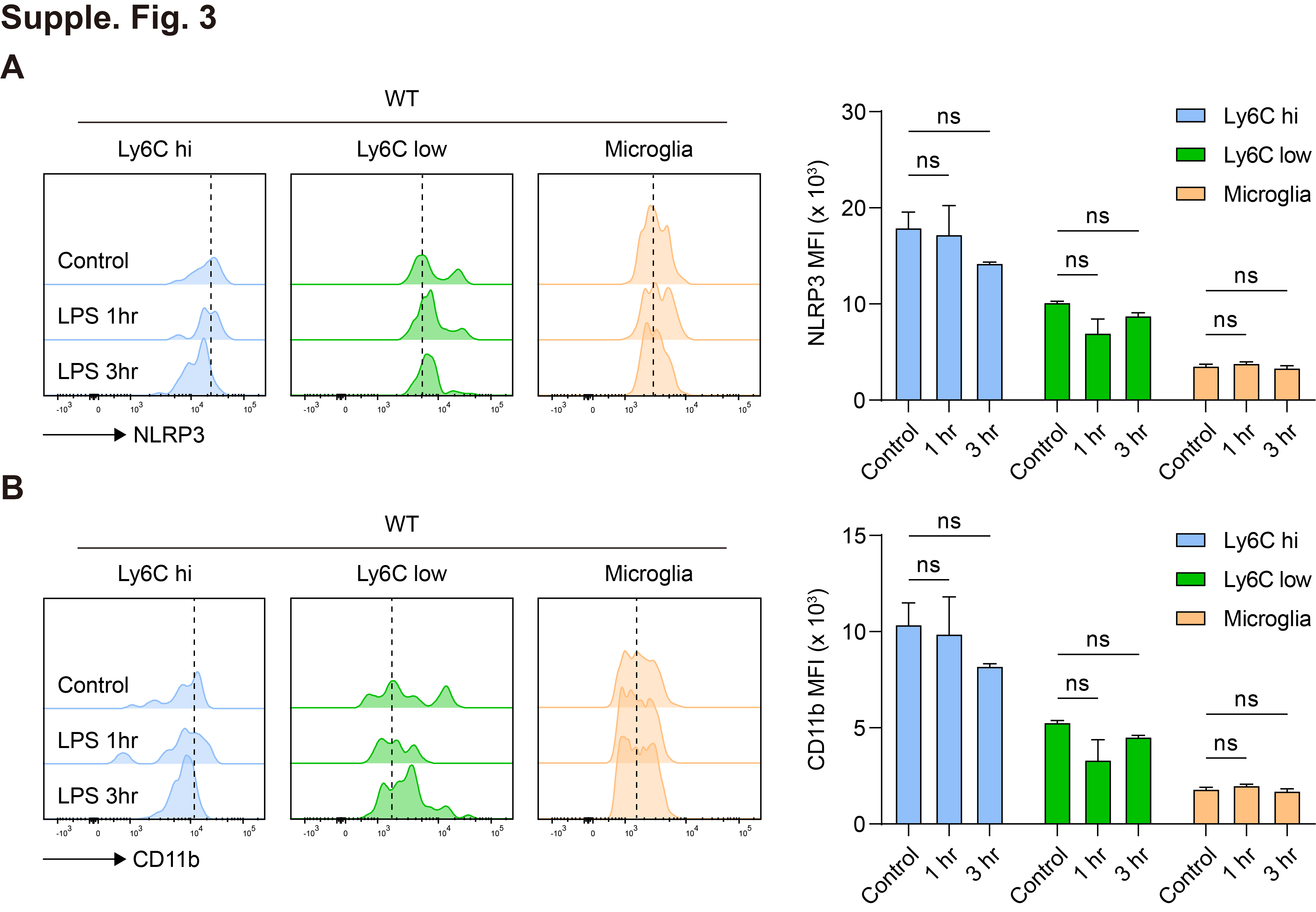

Supplement: Supplementary file 4 — Supplementary Material 4 [file 12974_2025_3468_MOESM4_ESM.png]

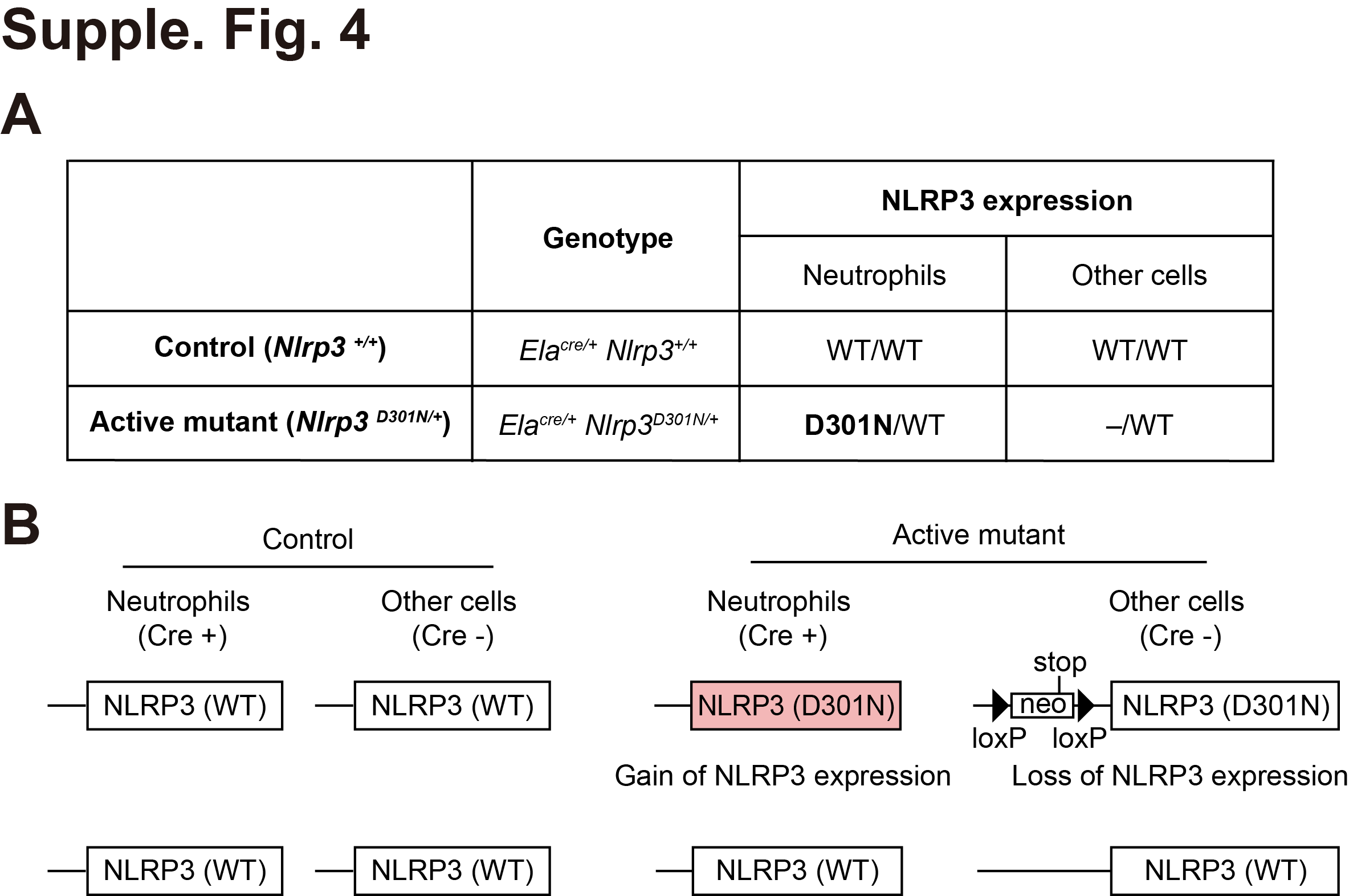

Supplement: Supplementary file 5 — Supplementary Material 5 [file 12974_2025_3468_MOESM5_ESM.png]

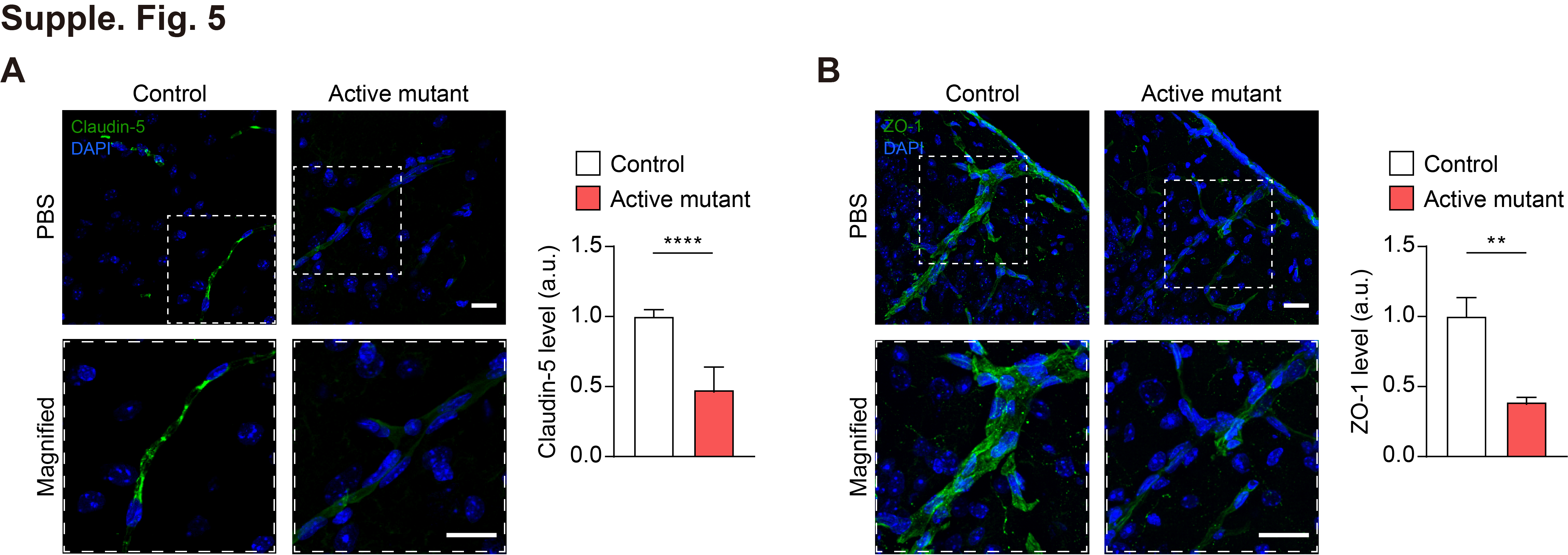

Supplement: Supplementary file 6 — Supplementary Material 6 [file 12974_2025_3468_MOESM6_ESM.png]

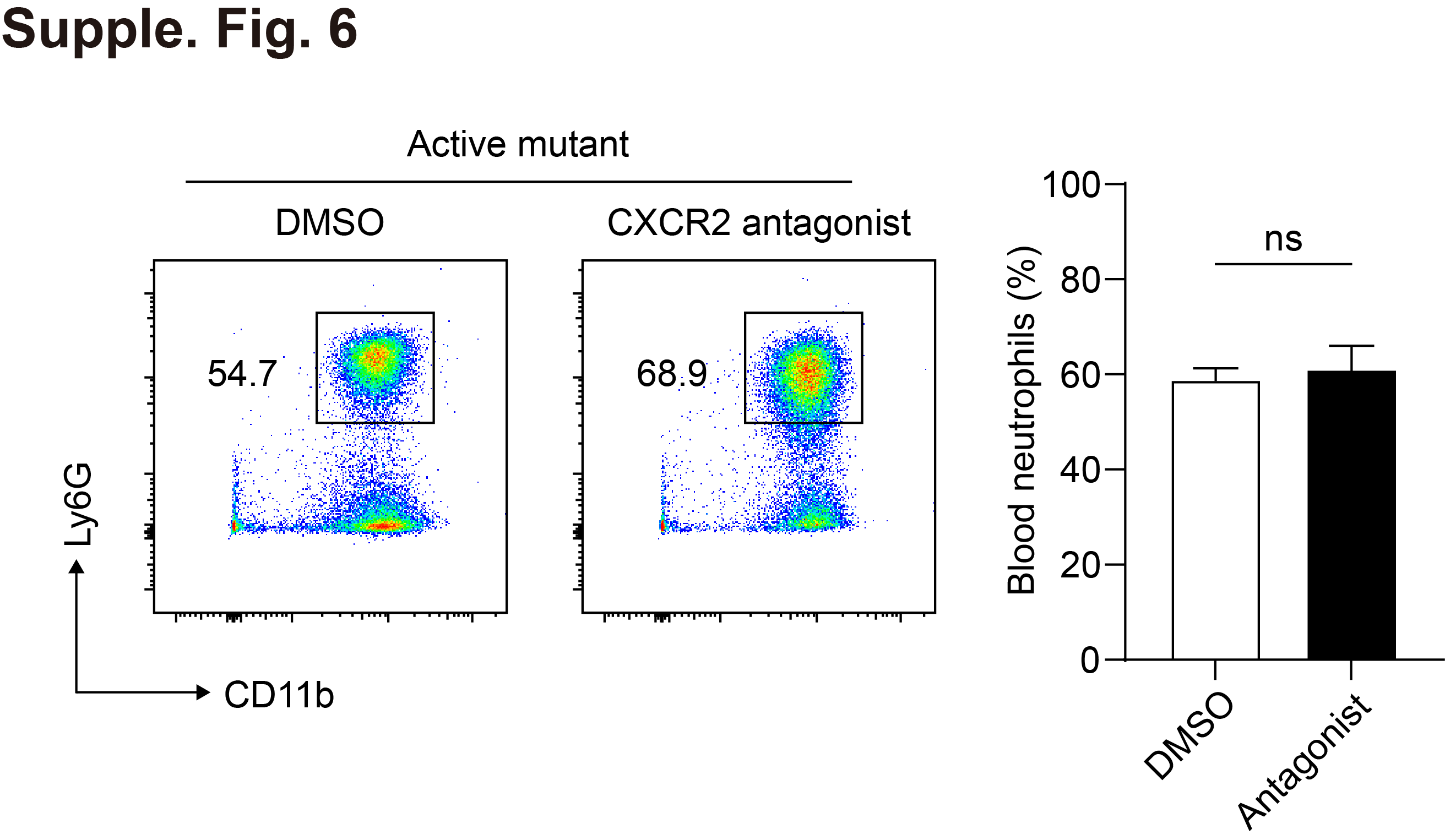

Supplement: Supplementary file 7 — Supplementary Material 7 [file 12974_2025_3468_MOESM7_ESM.png]
